# Association Between Ratio of Triglyceride to High-Density Lipoprotein Cholesterol and Cardiovascular and All-Cause Mortality in Non-Diabetic Hemodialysis Patients

**DOI:** 10.3390/medsci13040272

**Published:** 2025-11-15

**Authors:** Jane Pitanupong, Arunchai Chang, Apichai Wattanapisit

**Affiliations:** 1 Division of Nephrology, Department of Internal Medicine, Thasala Hospital, Nakhon Si Thammarat 80160, Thailand; janetleman4@hotmail.com; 2 Division of Gastroenterology, Department of Internal Medicine, Hatyai Hospital, Songkhla 90110, Thailand; busmdcu58@gmail.com; 3 Department of Clinical Medicine, School of Medicine, Walailak University, Nakhon Si Thammarat 80160, Thailand; 4 Family Medicine Clinic, Walailak University Hospital, Nakhon Si Thammarat 80160, Thailand; 5 The Excellent Center of Community Health Promotion, Walailak University, Nakhon Si Thammarat 80160, Thailand

**Keywords:** hemodialysis, high-density lipoprotein cholesterol, non-diabetic, survival, triglyceride

## Abstract

This study examined whether the triglyceride-to-high-density lipoprotein cholesterol (TG/HDL-C) ratio is associated with cardiovascular and all-cause mortality among non-diabetic patients undergoing hemodialysis. From June 2017 to December 2023, patients followed until December 2024 were categorized into two groups based on their baseline TG/HDL-C ratio: those with a high TG/HDL-C ratio (>3.29) and those with a non-elevated TG/HDL-C ratio (≤3.29). The association between TG/HDL-C ratio and CV and all-cause mortality was examined by univariate and multivariate Cox regression analyses. Of the 138 patients, 43 were categorized into the high TG/HDL-C ratio group and 95 into the non-elevated TG/HDL-C ratio group. The non-elevated TG/HDL-C ratio group had significantly increased cardiovascular survival rates of 1, 3, and 5 years (97.8% vs. 85.2%, 96.2% vs. 70.0%, and 87.0% vs. 52.2%, respectively; *p* < 0.05) and overall survival rates of 1, 3, and 5 years (95.8% vs. 79.1%, 89.6% vs. 62.9%, and 73.9% vs. 40.7%, respectively; *p* < 0.05). In the proportional hazards model, a high TG-HDL ratio was an independent predictor of cardiovascular mortality (hazard ratio [HR]: 6.799; 95% confidence interval [CI]: 2.276–20.313; *p* = 0.001) and all-cause mortality (HR: 2.88; 95% CI: 1.16–7.17; *p* = 0.023). A high TG/HDL-C ratio was associated with CV and overall mortality in non-diabetic HD patients. Further research will be required to explore changes in the serum TG/HDL-C ratio, assess lipoprotein profiles, and determine their outcomes in this group.

## 1. Introduction

The number of individuals with chronic kidney disease (CKD) requiring hemodialysis (HD) in Thailand has been rising significantly, driven by the rising prevalence of CKD and end-stage kidney disease (ESKD) [1]. Among these patients, cardiovascular disease (CVD) is a leading cause of mortality, contributing to nearly half of all deaths.

Among patients with CKD, low-density lipoprotein cholesterol (LDL-C) is an insufficient marker for cardiovascular risk assessment because its association with coronary events is limited in those with a low estimated glomerular filtration rate [2]. Furthermore, studies involving HD patients have failed to demonstrate any clear cardiovascular benefits from aggressive strategies aimed at reducing LDL-C levels [3]. Elevated serum triglyceride (TG) levels and reduced high-density lipoprotein cholesterol (HDL-C) levels are recognized as independent cardiovascular risk factors in the general population, regardless of LDL-C levels [4]. Identifying simple, cost-effective markers for this high-risk population is crucial. The TG/HDL-C ratio is a readily available marker for screening and risk stratification of cardiovascular complications in HD patients.

A high TG/HDL-C ratio is considered a strong indicator of the presence of atherogenic small LDLs [5,6]. In line with this, a previous study among HD patients found that a TG/HDL-C ratio > 3.29 was strongly correlated with an increased risk of death [7]. Therefore, our study adopted this empirically validated threshold to categorize patients [6,7]. Insulin resistance is known to cause atherogenic dyslipidemia, characterized by increased serum TG concentrations and decreased HDL-C concentrations. This metabolic shift is precisely why the TG/HDL-C ratio is involved in decreased insulin secretion and poor glycemic control among Type 2 DM patients [8]. Previous studies demonstrated the association between a high TG/HDL-C ratio and DM in the prediction of CV mortality [5,9].

While the TG/HDL-C ratio has been explored in the general HD population, the predictive value in these cohorts is often confounded by the high prevalence of Type 2 DM. DM and its associated insulin resistance independently influence the TG/HDL-C ratio. This metabolic interaction may obscure the direct prognostic value of the ratio itself. Consequently, a specific research gap exists regarding the independent association of the TG/HDL-C ratio with mortality in HD patients without the confounding influence of diabetes. Therefore, this study aimed to address this gap by focusing exclusively on a non-diabetic HD cohort.

Therefore, this study aimed to investigate whether the baseline TG/HDL-C ratio, measured at the initiation of HD, is associated with cardiovascular and all-cause mortality in non-diabetic HD patients, using a predefined cut-off of >3.29 for risk categorization.

## 2. Methods

### 2.1. Study Design

This retrospective cohort study obtained patient information between June 2017 and December 2023 at Thasala Hospital, a general hospital located in Southern Thailand. The study was based on the medical records of outpatients at the Hemodialysis Center in Thasala Hospital. The study protocol was approved by the Institutional Review Board of the Nakhon Si Thammarat Provincial Health Office (NSTPH 170/2567, approval date: 29 October 2024) and was carried out in accordance with the Declaration of Helsinki. Written informed consent was not required, as the study was retrospective in design and patient information was anonymized prior to analysis. Despite the coincidence of the study period with the COVID-19 pandemic, no significant disruptions in dialysis care related to the pandemic were observed among the study participants.

### 2.2. Patient Recruitment and Data Collection

We enrolled adult patients (age ≥ 18 years) with ESKD treated with HD as the primary kidney replacement therapy (KRT). For this study, ‘non-diabetic patients’ were operationally defined as individuals with no prior clinical diagnosis of Type 1 or Type 2 diabetes mellitus (DM) and whose cause of ESKD was not attributed to diabetic nephropathy. The exclusion criteria were as follows: (1) age < 18 years, (2) diagnosed with DM or diabetic nephropathy before HD, (3) change in KRT to peritoneal dialysis or renal transplantation, (4) survival less than 3 months following the initiation of HD, (5) concomitant severe cerebral or cardiopulmonary dysfunction, and (6) insufficient information (e.g., TG, HDL-C, loss to follow-ups).

Patient data were retrospectively collected from electronic medical records (EHRs). The presence of comorbidities, such as hypertension, ischemic heart disease, and cerebrovascular disease, was ascertained by a thorough review of the patients’ EHRs and validated against physician notes. For this study, ‘time zero’ for the survival analysis was defined as the date of HD initiation. The ‘baseline’ demographic and clinical variables—including age, sex, body mass index, the aforementioned comorbidities, medication usage, etiology of ESKD, clinical hypervolemia, unscheduled HD, and medications, including erythropoietin—were all recorded as of the initiation date. Baseline biochemical data (hemoglobin, FBS, total cholesterol (TC), TG, HDL-C, LDL-C, electrolytes, calcium (Ca), phosphate (P), and albumin (Alb)) were defined as the measurements taken within one week of HD initiation, drawn from a fasting state before a dialysis session. According to the policy on erythropoietin administration, patients with chronic kidney disease who were not receiving kidney replacement therapy were excluded from the Universal Coverage Scheme package. Based on patients’ preferences, many patients in this study denied receiving erythropoietin before HD.

### 2.3. Definitions and Outcomes

An elevated TG/HDL-C ratio > 3.29 was demonstrated to correlate with increased risk of death in HD patients [7]. Accordingly, patients in the present study were stratified into two groups based on their TG/HDL-C ratio at the initiation of HD: a high TG/HDL-C group (TG/HDL-C ratio > 3.29) and a low TG/HDL-C group (TG/HDL-C ratio ≤ 3.29).

The primary and secondary outcomes were cardiovascular and overall survival, respectively. Cardiovascular-related mortality included deaths due to myocardial infarction, unstable angina, cardiomyopathy, arrhythmia, heart failure, cardiac arrest, cerebrovascular disease, or peripheral vascular disease. Causes of death were obtained from medical records or follow-up and confirmed by physicians or the HD follow-up panel. Survival was defined as the time from the date of diagnosis of HD until death or last follow-up, and overall mortality was defined as death due to any cause.

### 2.4. Statistical Analysis

Categorical variables are presented as frequencies and percentages, and Pearson’s chi-square or Fisher’s exact tests were used to assess significant differences. Continuous variables are presented as means with standard deviations (SDs) or medians with interquartile ranges (IQRs) and tested for significance using *t*-test or Wilcoxon rank-sum tests. Survival analysis was performed using the Kaplan–Meier method, and the log-rank test was used to analyze the statistical differences between variables. The normality of continuous variables was assessed using the Shapiro–Wilk test and visual inspection of histograms. Skewed data were log-transformed as appropriate. For Cox regression analyses, linearity and proportional hazards assumptions were verified using residual-based diagnostics. The Cox proportional hazards model was used to identify the variables influencing survival. Variables associated with outcomes in the univariate analysis with a *p*-value < 0.1 were considered candidates for the multivariate Cox proportional hazards models. We utilized a forward stepwise selection method (with *p* < 0.05 for entry and *p* > 0.10 for removal) to identify the most robust independent predictors. Only covariates that remained statistically significant (*p* < 0.05) in the final iterative model were retained and presented. Additionally, a Receiver Operating Characteristic (ROC) curve analysis was conducted to determine the optimal threshold for the TG/HDL-C ratio in predicting cardiovascular mortality within this cohort. The Youden index was used to determine the optimal cut-off point. To validate the predictive strength of the TG/HDL-C ratio, we also performed comparative Cox regression analyses for other established lipid ratios, including the total cholesterol/HDL ratio and LDL/HDL ratio. In the primary analysis, TG/HDL-C was dichotomized at the pre-specified cut-off value of 3.29 to assess its association with cardiovascular mortality. Finally, model stability was assessed by checking for multicollinearity among the final predictors. Variance Inflation Factors (VIFs) were calculated for the covariates retained in the final multivariate models. All VIFs were found to be <1.1, indicating that multicollinearity was not a significant concern. All data analyses were performed using the statistical program Stata Version 15.1 (StataCorp LLC, College Station, TX, USA). Statistical significance was set at a *p* < 0.05.

## 3. Results

### 3.1. Patient Population

**Figure 1:**
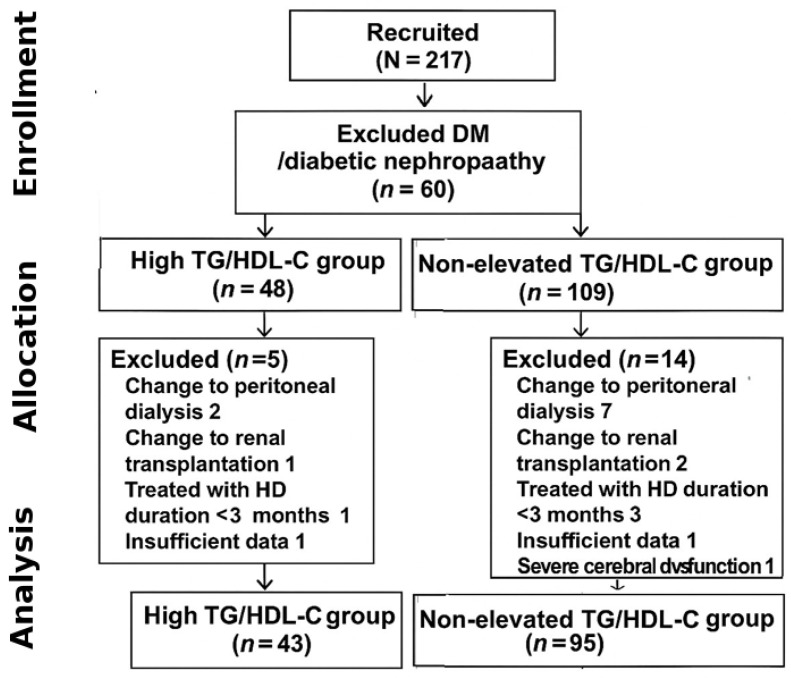
Flow of enrollment.

A total of 217 patients were initially recruited, and 79 patients were excluded from this study; 60 patients were excluded due to diagnosis as DM or diabetic nephropathy before HD, 12 patients were excluded due to a change in KRT (9 patients switched to peritoneal dialysis and 3 patients switched to renal transplantation), 2 patients had insufficient data for analysis, 4 patients had been treated with HD for less than 3 months, and 1 patient had severe cerebral dysfunction. The final analytic cohort comprised 138 patients (43 in the high TG/HDL-C group and 95 in the non-elevated group). The detailed patient selection process is shown in Figure 1. Therefore, 138 patients were included in the analyses, comprising 56 (40.6%) males and an average age of 60.5 ± 15.3 years. Overall, the median follow-up time was 26.6 months. The etiologies of ESKD were hypertensive nephrosclerosis in 110 (79.7%) patients, chronic glomerulonephritis in 11 (7.9%) patients, and obstructive uropathy in 3 (2.2%) patients.

According to the TG/HDL-C ratio, 43 (31%) patients were classified as the high TG/HDL-C group, while 95 (69%) were classified as the non-elevated TG/HDL-C group. Comparisons of the demographic data are shown in Table 1. There were no significant differences between the two groups in terms of sex, age, body mass index, etiology of kidney failure, medication use, fasting blood sugar, total cholesterol, low-density lipoprotein (LDL) levels, calcium, and phosphate levels. However, patients in the high TG/HDL-C group were significantly more likely to have a history of ischemic heart disease compared to those in the non-elevated TG/HDL-C group (37.2% vs. 16.8%, *p* = 0.016). Use of calcium supplements was significantly lower in the high TG/HDL-C group compared to the low TG/HDL-C group (30.9% vs. 60.0%, *p* = 0.02).

### 3.2. Survival Analysis

**Figure 2:**
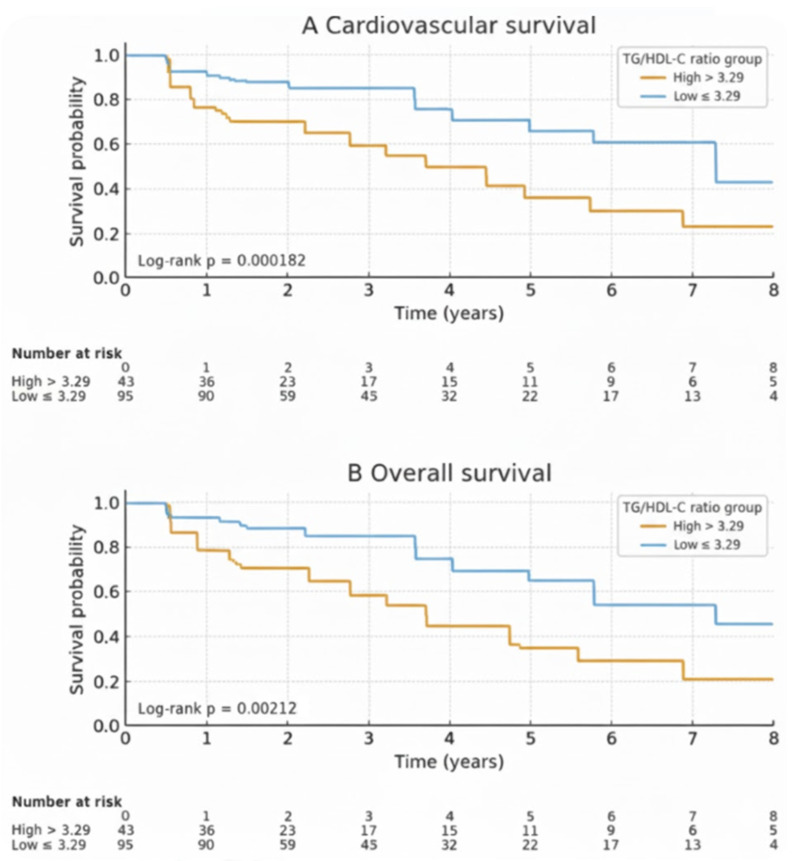
Comparison of cumulative cardiovascular survival (**A**) and overall survival (**B**) rates. The survival rate of HD patients with a TG/HDL-C ratio ≤ 3.29 (blue line) was higher compared to those with a TG/HDL-C ratio > 3.29 (yellow line) for both cardiovascular survival and overall survival.

During the follow-up period, a total of 44 deaths (31.9%) occurred among patients. Of these, 28 deaths (63.6%) were attributed to cardiovascular causes, and 10 deaths (22.7%) were due to sepsis. The cumulative cardiovascular survival rates at 1, 3, and 5 years were 94.7%, 87.2%, and 75.6%, respectively. The cumulative overall survival rates at 1, 3, and 5 years were 91.9%, 80.1%, and 65.2%, respectively. Kaplan–Meier analysis revealed that patients in the high TG/HDL-C ratio group had lower cardiovascular and overall mortality-free survival rates compared to the non-elevated group (Figure 2). The cumulative cardiovascular and overall mortality-free survival rates, along with the hazard ratios derived from Cox proportional hazards models, are presented in Table 2. Patients in the non-elevated TG/HDL-C group (≤3.29) had higher cardiovascular survival rates than those in the high TG/HDL-C group (>3.29). The 1-, 3-, and 5-year cumulative cardiovascular survival rates were 97.8% vs. 85.2%, 96.2% vs. 70.0%, and 87.0% vs. 52.2%, respectively (HR = 3.58; 95% CI: 1.69–7.60; log-rank: *p* = 0.0019). Similarly, overall survival was significantly better in the non-elevated TG/HDL-C group. The 1-, 3-, and 5-year cumulative overall survival rates were 95.8% vs. 79.1%, 89.6% vs. 62.9%, and 73.9% vs. 40.7%, respectively (HR = 2.62; 95% CI: 1.48–4.63; log-rank: *p* = 0.0027).

### 3.3. Predictors of Survival

The Cox proportional hazards model analysis results for cardiovascular survival according to the primary outcome are summarized in Table 3. In the univariate analysis, the risk factors significantly associated with an elevated cardiovascular-related mortality risk included age, hypertension, underlying cerebrovascular disease, hemoglobin level, albumin, and a TG/HDL-C ratio greater than 3.29. In the multivariate analysis, underlying cerebrovascular disease (HR: 3.662; 95% CI: 1.058–12.678; *p* = 0.041), each 1 mg/dL increase in serum albumin (HR: 0.339; 95% CI: 0.166–0.696; *p* = 0.003), and a TG/HDL-C ratio >3.29 (HR 6.799; 95% CI: 2.276–20.313; *p* = 0.001) were independently associated with increased cardiovascular mortality. To further evaluate the prognostic performance of other lipid ratios, we additionally compared the hazard ratios of TG/HDL-C, TC/HDL-C, LDL/HDL-C, and non-HDL-C with cardiovascular mortality. The results are summarized in Supplementary Table S2.

Table 4 presents the results of the Cox proportional hazards model analysis for overall survival. In the univariate analysis, the risk factors significantly associated with an elevated all-cause mortality risk were underlying ischemic heart disease, cerebrovascular disease, hemoglobin level, albumin, and a TG/HDL-C ratio > 3.29. After adjusting for confounding factors, every 1 mg/dL increase in serum albumin (HR 0.48; 95% CI: 0.28–0.83; *p* = 0.008) and a TG/HDL-C ratio > 3.29 (HR 2.88; 95% CI: 1.16–7.17; *p* = 0.023) were independently associated with increased all-cause mortality in the multivariate analysis.

**Figure 3:**
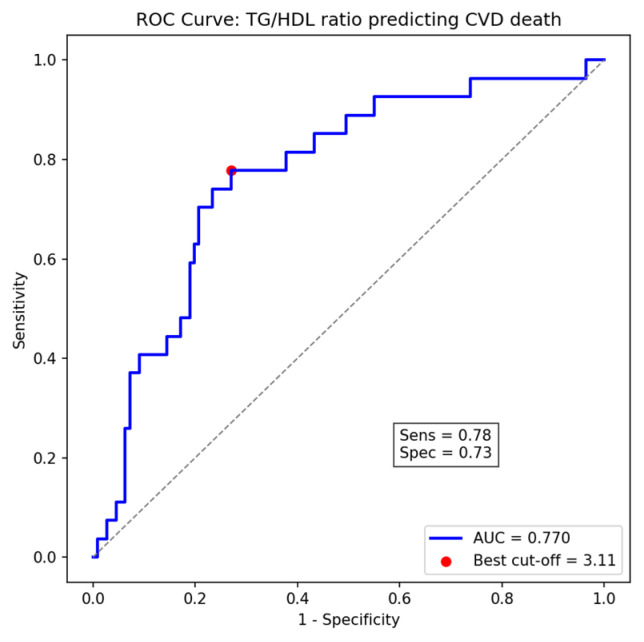
Receiver Operating Characteristic (ROC) curve of TG/HDL-C ratio for predicting cardiovascular death, showing the optimal cut-off point (3.11) determined by Youden’s index (AUC = 0.77).

ROC curve analysis revealed that the TG/HDL-C ratio exhibited a moderate ability to predict cardiovascular mortality, with an area under the curve (AUC) of 0.77 (95% CI: 0.63–0.79). The optimal cut-off value identified using Youden’s index was 3.11, providing a sensitivity of 78% and specificity of 73%, which closely approximates the pre-specified threshold of 3.29 used for Cox regression analyses (Figure 3).

## 4. Discussion

The main finding of this retrospective cohort study is that a high baseline TG/HDL-C ratio (>3.29) was an associated factor of both cardiovascular-related and all-cause mortality in incident non-diabetic HD patients. After adjustment for confounders, a high TG/HDL-C ratio remained significantly associated with increased cardiovascular-related mortality after HD. The imbalance in ischemic heart disease between TG/HDL-C groups was adjusted in the multivariate Cox models; however, residual confounding cannot be fully excluded, and the number of cardiovascular events was relatively limited. Therefore, this relationship should be interpreted cautiously as a statistical association rather than a definitive independent effect.

We acknowledge that the study period overlapped with the COVID-19 pandemic, a potential confounding factor. Upon review of mortality data, we identified three deaths specifically attributed to COVID-19. Of these cases, one patient was in the high TG/HDL ratio group, and two patients were in the non-elevated TG/HDL ratio group.

Although dyslipidemia is widely recognized as a conventional risk factor for cardiovascular disease in the general population, serum LDL-C does not consistently reflect cardiovascular risk in patients with ESKD [10]. Lowering LDL-C with statins does not translate into meaningful reductions in cardiovascular events or mortality among patients receiving HD [11]. Insulin resistance was found to be a risk of a high TG/HDL-C ratio [12]. It has also been linked to reduced insulin secretion and impaired glycemic control in individuals with type 2 DM [13]. An interaction between the TG/HDL-C ratio and DM status in predicting outcomes among HD patients was observed in previous studies [5,14]. Also, this study focused exclusively on non-diabetic HD patients.

In this retrospective cohort of 138 HD patients, we found that a high TG/HDL-C ratio was associated with cardiovascular-related and overall mortality. The observed association between a high TG/HDL-C ratio and cardiovascular mortality in our cohort is biologically plausible. This relationship may be explained by several interrelated metabolic and vascular mechanisms, as described below. A higher TG/HDL-C ratio has been linked to increased small dense LDL-C particle levels, which are recognized as highly atherogenic [15,16]. Small dense LDL-C particles have a lower affinity for LDL receptors and a longer residence time in circulation [17]. Moreover, their larger specific surface area not only facilitates easier penetration into the arterial wall but also strengthens their binding affinity to arterial wall proteoglycans [18]. Previous studies showed that small dense LDL-C was the best marker for predicting carotid arteriosclerosis and progression of arterial stiffness, independent of other cardiovascular risk factors [19,20]. Otherwise, a high TG/HDL-C ratio may have adverse effects through endothelial dysfunction [21], chronic low-grade inflammation [22], increased oxidative stress, such as interleukin-6 [23], and abnormalities in fibrinolysis and coagulation [24], contributing to the development and progression of arteriosclerosis.

In the present study, a TG/HDL-C ratio >3.29 was significantly associated with poorer cardiovascular and overall survival in HD patients. This finding contrasts with that of Chang et al. [25], who reported a paradoxical protective effect at a higher cut-off value (>3.64). A key difference is that our study included only non-diabetic patients, who generally had lower triglyceride levels, making the threshold more sensitive to adverse lipid profiles. In contrast, Chang’s study included diabetic patients with higher TG levels, and the paradoxical result may partly reflect the malnutrition–inflammation complex frequently seen in dialysis populations, where lower TG/HDL-C ratios may indicate protein-energy wasting and inflammation, while higher ratios suggest better nutritional reserves. Ethnic and dietary background, dialysis practices, and analytic approaches may also contribute to these discrepancies. Moreover, our cohort consisted of incident HD patients, while Chang et al. analyzed prevalent cases, which may represent different stages of metabolic adaptation. We assessed baseline TG/HDL-C ratios, whereas some prior studies used time-varying lipid values, which may have captured different temporal effects. Differences in outcome definitions—such as cardiovascular mortality versus composite cardiovascular events—may further explain variations in hazard estimates. Importantly, our findings align with those of Chen et al. [5] and Hasegawa et al. [9], who both demonstrated that elevated TG/HDL-C ratios are associated with increased cardiovascular risk in CKD and hemodialysis patients.

In addition, our ROC analysis yielded an optimal cut-off value of 3.11, determined by the Youden index, which closely approximates the previously validated threshold of 3.29. This supports the robustness of the TG/HDL-C ratio as a prognostic indicator and suggests that the previously established cut-off is largely applicable to our population, with minor variations likely reflecting population-specific metabolic characteristics. In this study, we further compared the predictive value of TG/HDL-C with other lipid ratios using the pre-specified threshold of 3.29 to maintain consistency with group definitions throughout the manuscript. The AUC (0.77) was derived from time-dependent ROC analysis to assess overall discriminatory ability, while the Cox regression analyses using the categorical cut-off (3.29) quantified the associated risk. As shown in Supplementary Table S2, the TG/HDL-C ratio demonstrated the strongest association with cardiovascular mortality (HR = 1.25, 95% CI 1.05–1.45, *p* = 0.015) compared to the TC/HDL ratio and the LDL/HDL ratio. Furthermore, when triglyceride and HDL-C levels were analyzed individually, TG was positively associated with cardiovascular mortality (HR = 1.02 per 10 mg/dL); however, the TG/HDL-C ratio yielded a stronger and more consistent association than either component alone. This finding strengthens the argument that the TG/HDL-C ratio, reflecting atherogenic dyslipidemia, may be a superior prognostic marker in this non-diabetic hemodialysis population.

In supplementary Kaplan–Meier analyses, higher triglyceride levels (>150 mg/dL) were associated with lower cardiovascular mortality-free survival but not with overall mortality-free survival, whereas lower HDL-C levels (<40 mg/dL) were related to reduced overall mortality-free survival but not to cardiovascular mortality-free survival (Supplementary Figures S1 and S2). These findings suggest that TG and HDL-C individually influence different dimensions of mortality risk. At the same time, their combined ratio (TG/HDL-C) provides a more integrated indicator of atherogenic dyslipidemia and survival outcomes in hemodialysis patients. This study found that low albumin levels were independently associated with both cardiovascular and all-cause mortality in hemodialysis patients. Serum albumin not only is a crucial marker of nutritional status but also reflects inflammation and fluid balance. Hypoalbuminemia has been linked to intradialytic hypotension [26] and progressive left ventricular dysfunction [27]. Furthermore, previous studies have demonstrated associations between hypoalbuminemia and increased levels of inflammatory cytokines and oxidative stress markers, including interleukin-6, high-sensitivity C-reactive protein (hs-CRP), and tumor necrosis factor-α, all of which are established risk factors for atherosclerotic vascular disease in HD patients [28,29]. In addition, hypoalbuminemia has been correlated with increased carotid intimal thickness and arterial stiffness, as measured by pulse wave velocity, both of which are strongly associated with cardiovascular mortality [30,31,32]. Serum albumin serves as a comprehensive prognostic marker in the dialysis population, reflecting the combined impact of malnutrition, inflammation, and vascular pathology. Although multicollinearity between the TG/HDL-C ratio and serum albumin was not observed (VIFs ≈ 1.0), these two parameters may still reflect related biological pathways. Low albumin levels typically indicate malnutrition and inflammation, which are also linked to dyslipidemia and higher TG/HDL-C ratios. Therefore, TG/HDL-C and serum albumin might represent complementary components of the malnutrition–inflammation–atherosclerosis complex contributing to cardiovascular risk in HD patients.

This study showed that underlying cerebrovascular disease before HD was associated with cardiovascular-related mortality. A previous study revealed that a history of stroke prior to dialysis initiation was associated with cardiovascular-related survival of HD patients [33], which may be explained by the fact that cerebrovascular disease may reflect atherosclerosis, and vascular calcification deteriorates during dialysis [33,34].

This study has some limitations. Firstly, it was a single-center retrospective observational study with a relatively small sample size. Consequently, our study may have been underpowered to detect smaller, yet potentially clinically relevant, effect sizes or associations, as it exclusively comprised Thai patients, which may limit the generalizability of the findings to other populations. Further validation from larger, multi-center studies is warranted to confirm the generalizability and robustness of our results. Secondly, we excluded patients who died or changed kidney replacement therapy modality within the first three months after HD initiation. Although this approach ensured a more stable dialysis cohort, it may have introduced selection bias by removing early deaths and modality changes, potentially overestimating survival and hazard ratios. Thirdly, the number of cardiovascular events was relatively small, which limits statistical power and may have affected the stability of the multivariable Cox model. Additionally, residual confounding from unmeasured dialysis-related factors (e.g., dialysis adequacy, vascular access, and residual renal function) cannot be ruled out. The timing of baseline TG/HDL-C measurement, defined as the most recent lipid profile obtained before HD initiation, may also introduce uncertainty, as temporal variation and changes during follow-up were not evaluated. Moreover, because this study was retrospective in nature, causal inference cannot be established. Non-cardiovascular deaths were treated as censored observations, which does not fully account for competing risks and may overestimate hazard ratios for cardiovascular mortality. Finally, we lacked data on several traditional and non-traditional cardiovascular risk factors, such as high-sensitivity C-reactive protein, subjective global assessment, and specific lipoprotein subfractions (e.g., apolipoprotein B or small dense LDL-C), which might further clarify the mechanisms linking TG/HDL-C to cardiovascular outcomes.

In summary, this single-center retrospective study demonstrated that, among non-diabetic patients with ESKD undergoing HD, a higher TG/HDL-C ratio (>3.29) at the initiation of HD was significantly associated with cardiovascular and all-cause mortality. These results indicate that the TG/HDL-C ratio could function as a straightforward and accessible marker for cardiovascular risk stratification in this population. Further multi-center and prospective studies are warranted to confirm these associations, evaluate longitudinal changes in TG/HDL-C, and clarify the underlying lipoprotein and inflammatory mechanisms.

## Figures and Tables

**Table 1:** Baseline demographic data and clinical characteristics of the two groups.

Characteristic	High TG/HDL-C Group (*n* = 43)	Non-Elevated TG/HDL-C Group (*n* = 95)	*p*-Value
Male sex	20 (46.5%)	46 (48.4%)	0.981
Age (years): mean (SD)	63.51(14.50)	60.27 (15.77)	0.240
BMI (kg/m^2^): median (IQR)	23.85 (20.63, 27.43)	23.18 (20.41, 25.32)	0.306
Systolic blood pressure: (mmHg): mean (SD)	147.88 (26.20)	152.15 (28.96)	0.433
Diastolic blood pressure: (mmHg): median (IQR)	75.50 (63.5, 84)	79 (67.2, 90.8)	0.110
Pulse pressure: median (IQR)	63.00 (52.00, 84.50)	72.00 (56.50, 84.50)	0.296
Smoking	10 (23.3%)	21 (22.1%)	1.000
Unscheduled HD	14 (32.6%)	43 (45.3%)	0.344
Comorbidity			
Hypertension	39 (90.7%)	88 (92.6%)	0.961
Dyslipidemia	32 (74.4%)	66 (69.5%)	0.696
Ischemic heart disease	16 (37.2%)	16 (16.8%)	**0.016**
Cerebrovascular disease	8 (18.6%)	12 (12.6%)	0.508
Peripheral arterial disease	2 (4.7%)	5 (5.3%)	0.785
Etiology of kidney failure			0.778
Hypertension	35 (81.4%)	76 (80.0%)	
Chronic glomerulonephritis	2 (4.7%)	9 (9.6%)	
HCV-induced GN	0	1 (1.1%)	
Obstructive uropathy	1 (2.3%)	2 (2.1%)	
Unknown	3 (7.0%)	3 (3.2%)	
Medication			
Aspirin	20 (46.5%)	30 (31.6%)	0.173
Clopidogrel	9 (20.9%)	8 (8.4%)	0.270
Calcium channel blocker	37(86.0%)	84 (88.4%)	0.248
Angiotensin-converting enzyme inhibitors	2 (4.7%)	4 (4.2%)	1.000
Angiotensin-receptor blocker	5 (11.6%)	17 (17.9%)	0.496
Statin	29 (64.3%)	66 (67.3%)	1.000
Spironolactone	1 (2.3%)	4 (4.2%)	0.537
Calcium supplementation	15 (34.9%)	57 (60.0%)	**0.021**
Vitamin D3	3 (6.9%)	19 (20.0%)	0.465
Laboratory			
Fasting blood sugar (mg/dL): mean (SD)	96.56 (14.30)	94 (12.25)	0.369
Hemoglobin (g/dL): median (IQR)	8.20 (7.50, 10.05)	8.40 (7.60,9.60)	0.818
BUN (mg/dL): median (IQR)	84.00 (59.50, 109.80)	87.90 (65.00,106.50)	0.863
Total cholesterol (mg/dL): median (IQR)	175.00 (143.5, 225.0)	169.0 (147.0, 202.0)	0.578
TG (mg/dL): median (IQR)	186.0 (142.0, 228.0)	102.0 (73.0, 124.5)	**0.001**
HDL (mg/dL): median (IQR)	38.0 (34.4, 44.6)	55.0 (47.6, 68.0)	**0.001**
LDL (mg/dL): median (IQR)	97.00 (68.85, 140.80)	90.8(69.28, 112.75)	0.578
Serum calcium (mg/dL): median (IQR)	8.80 (8.4, 9.25)	8.80 (8.10, 9.30)	0.708
Serum phosphate (mg/dL): median (IQR)	5.50 (4.20, 6.70)	5.10 (4.10, 6.90)	0.762
Serum albumin (mg/dL): median (IQR)	3.34(0.8)	3.43 (0.75)	0.527

Data are expressed as numbers (%) unless specified. Bold *p*-values show statistical significance. BMI: body mass index; BUN: blood urea nitrogen; GN: glomerulonephritis; IQR: interquartile range; HCV: hepatitis C virus; HDL: high-density lipoprotein; LDL: low-density lipoprotein; SD: standard deviation; TG: triglyceride. *p*-values were calculated using Pearson’s chi-square or Fisher’s exact test for categorical variables, and *t*-test or Wilcoxon rank-sum test for continuous variables as appropriate.

**Table 2:** Cardiovascular and overall mortality-free survival between TG/HDL-C ratio groups, with hazard ratios derived from Cox proportional hazards models.

	Non-Elevated TG/HDL-C Ratio Group (*n* = 95)	High TG/HDL-C Ratio Group (*n* = 43)	HR (Cox Model, 95% CI)	*p*-Value
Cardiovascular survival				
1-year survival	97.8	85.2		
3-year survival	96.2	70.0	3.58 (1.69–7.60)	0.0019
5-year survival	87.0	52.2		
Overall survival				
1-year survival	95.8	79.1		
3-year survival	89.6	62.9	2.62 (1.48–4.63)	0.0027
5-year survival	73.9	40.7		

CI: confidence interval; HDL-C: high-density lipoprotein cholesterol; HR: hazard ratio; TG: triglyceride.

**Table 3:** Univariate and multivariate Cox proportional hazard analyses for cardiovascular mortality.

Factors	Univariate			Multivariate		
	HR	(95% CI)	*p* Value	HR	(95% CI)	*p*-Value
Age, every 1 year increase	1.030	1.00–1.08	0.032			
Male sex	1.975	0.39–1.94	0.626			
BMI, every 1 kg/m^2^ increase	0.912	0.82–1.01	0.071			
Smoking	1.53	0.85–2.03	0.572			
Unscheduled HD	0.955	0.83–1.95	0.990			
Comorbidities						
Hypertension	1.66	1.21–2.30	0.018			
Dyslipidemia	1.41	0.53–3.75	0.496			
Ischemic heart disease	1.79	0.44–7.28	0.431			
Cerebrovascular disease	2.136	0.95–4.81	0.067	3.662	1.058–12.678	0.041
Peripheral arterial disease	2.13	0.35–5.18	0.513			
Medication						
Aspirin	1.62	0.75–3.51	0.217			
Clopidogrel	1.35	0.56–1.44	0.260			
Statin	1.05	0.28–3.89	0.943			
Calcium channel blocker	0.98	0.398–2.68	0.913			
Angiotensin-converting enzyme inhibitors	1.06	0.66–1.14	0.894			
Angiotensin-receptor blocker	1.03	0.63–1.17	0.923			
Spironolactone	1.28	0.30–5.45	0.741			
Calcium supplementation	3.05	0.40–23.48	0.285			
Vitamin D3	2.08	1.98–5.18	0.992			
Erythropoietin	0.97	0.39–2.42	0.941			
Laboratory						
Hemoglobin, every 1 g/dL decrease	1.39	1.02–1.91	0.044			
Serum albumin, every 1 mg/dL increase	0.36	0.19–0.66	0.010	0.339	0.166–0.696	0.003
BUN, every 10 mg/dL increase	1.03	0.93–1.04	0.600			
FBS, every 10 mg/dL increase	1.27	0.95–1.68	0.101			
Total cholesterol, every 10 mg/dL increase	0.98	0.95–1.007	0.284			
TG-HDL ratio > 3.29	4.55	1.84–11.20	0.010	6.799	2.276–20.313	0.001
TG, every 10 mg/dL increase	1.07	1.00–1.10	0.018			
HDL, every 10 mg/dL increase	0.72	0.54–1.10	0.059			
LDL, every 10 mg/dL increase	1.06	0.91–1.23	0.476			
Serum calcium, every 1 mg/dL increase	1.295	0.96–1.74	0.086			
Serum phosphate, every 1 mg/dL increase	1.06	0.64–1.45	0.961			

BMI: body mass index; BUN: blood urea nitrogen; FBS: fasting blood sugar; HD: hemodialysis; HDL: high-density lipoprotein; LDL: low-density lipoprotein; TG: triglyceride. Variables entered into the forward stepwise model (univariate *p* < 0.1) included those listed in the table. Hazard ratios (HRs) with 95% confidence intervals (CIs) were obtained from Cox proportional hazards regression models. Both univariate and multivariate results are presented; variables remaining statistically significant (*p* < 0.05) in the final model were retained.

**Table 4:** Univariate and multivariate Cox proportional hazards analyses for all-cause mortality.

Factors	Univariate			Multivariate		
	HR	(95% CI)	*p* Value	HR	(95% CI)	*p*-Value
Age, every 1 year increase	1.035	0.98–1.06	0.091			
Male sex	1.49	0.72–3.05	0.281			
BMI, every 1 kg/m^2^ increase	0.940	0.86–1.02	0.154			
Smoking	2.114	0.93–4.82	0.075			
Unscheduled HD	0.955	0.83–1.95	0.990			
Comorbidities						
Hypertension	1.36	1.1–1.44	0.104			
Dyslipidemia	1.59	0.70–3.65	0.270			
Ischemic heart disease	2.79	1.23–6.303	0.014			
Cerebrovascular disease	3.15	1.19–8.29	0.020			
Peripheral arterial disease	3.03	0.65–14.18	0.159			
Medication						
Aspirin	1.18	0.97–1.22	0.140			
Clopidogrel	1.25	0.67–1.43	0.404			
Statin	1.05	0.28–3.89	0.943			
Calcium channel blocker	0.50	0.20–1.26	0.141			
Angiotensin-converting enzyme inhibitors	4.60	0.81–26.14	0.085			
Angiotensin-receptor blocker	1.00	0.38–2.65	0.994			
Spironolactone	1.44	0.23–8.97	0.741			
Calcium supplementation	3.05	0.396–23.48	0.693			
Vitamin D3	0.31	0.10–1.96	0.421			
Erythropoietin use	0.58	0.25–1.32	0.190			
Laboratory						
Hemoglobin, every 1 g/dL decrease	1.107	0.92–1.32	0.026			
Serum albumin, every 1 mg/dL increase	0.454	0.27–0.76	0.022	0.48	0.28–0.83	0.008
BUN, every 10 mg/dL increase	1.009	0.92–1.11	0.600			
FBS, every 10 mg/dL increase	1.323	0.99–1.76	0.055			
Total cholesterol, every 10 mg/dL increase	0.962	0.89–1.037	0.847			
TG-HDL ratio > 3.29	3.358	1.48–7.62	0.004	2.88	1.16–7.17	0.023
TG, every 10 mg/dL increase	1.04	1.01–1.71	0.034			
HDL, every 10 mg/dL increase	0.89	0.72–0.98	0.027			
LDL, every 10 mg/dL increase	1.06	0.91–1.23	0.476			
Serum calcium, every 1 mg/dL increase	5.742	0.96–16.3	0.306			
Serum phosphate, every 1 mg/dL increase	1.245	0.94–1.34	0.104			

BMI: body mass index; BUN: blood urea nitrogen; FBS: fasting blood sugar; HD: hemodialysis; HDL: high-density lipoprotein; LDL: low-density lipoprotein; TG: triglyceride. Variables entered into the forward stepwise model (univariate *p* < 0.1) included those listed in the table. Hazard ratios (HRs) with 95% confidence intervals (CIs) were obtained from Cox proportional hazards regression models. Both univariate and multivariate results are presented; variables remaining statistically significant (*p* < 0.05) in the final model were retained.

## Data Availability

The original contributions presented in this study are included in the article/Supplementary Materials. Further inquiries can be directed to the corresponding author.

## Supplementary Materials

The following supporting information can be downloaded at: https://www.mdpi.com/article/10.3390/medsci13040272/s1; Table S1A. Multivariate Cox proportional hazards analyses for cardiovascular mortality; Table S1B. Multivariate Cox proportional hazards analyses for all-cause mortality; Table S2. Comparison of Hazard Ratios for Cardiovascular Mortality by Different Lipid Ratios (primary TG/HDL-C cut-off = 3.29); Figure S1. Kaplan–Meier curves showing cardiovascular and overall survival according to serum triglyceride levels (>150 mg/dL vs. ≤150 mg/dL); Figure S2. Kaplan–Meier curves showing cardiovascular and overall survival according to serum HDL-C levels (<40 mg/dL vs. ≥40 mg/dL).
