# Supplementary material for: Association Between Ratio of Triglyceride to High-Density Lipoprotein Cholesterol and Cardiovascular and All-Cause Mortality in Non-Diabetic Hemodialysis Patients
Source: Med Sci (Basel). 2025 Nov 15;13(4):272. doi: 10.3390/medsci13040272 (PMC12641832; doi:10.3390/medsci13040272)
Supplement: Supplementary file 1 [file medsci-13-00272-s001.zip › medsci-3963507-supplementary.pdf]

Supplementary Materials: Association Between Ratio of Triglyceride to High-Density Lipoprotein Cholesterol and Cardiovascular and All-Cause Mortality in Non-Diabetic Hemodialysis Patients

Supplementary Table S1A. Multivariate Cox proportional hazards analyses for cardiovascular mortality.

Factors	HR	95% CI	p-value
Age, every 1 year increase	1.015	0.985–1.045	0.333
BMI, every 1 kg/m^2 increase	0.402	0.132–1.223	0.109
Hypertension	0.309	0.063–1.530	0.150
Cerebrovascular disease	3.662	1.058 – 12.678	0.041
Serum albumin, every 1 mg/dL increase	0.339	0.166 – 0.696	0.003
Hemoglobin, every 1 g/dL decrease	1.131	0.950–1.346	0.165
Serum calcium, every 1 mg/dL increase	1.031	0.100–1.064	0.073
TG-HDL ratio >3.29	6.799	2.276 – 20.313	0.001

Supplementary Table S1B. Multivariate Cox proportional hazards analyses for all-cause mortality.

Factors	HR	95% CI	p-value
Age, every 1 year increase	1.011	0.987–1.034	0.373
Smoking	2.066	0.919–4.188	0.054
Ischemic heart disease	1.041	0.524–2.071	0.908
Cerebrovascular disease	0.492	0.188–1.285	0.147
ACEI	1.618	0.546–4.800	0.385
Hypertension	0.359	0.112–1.156	0.086
Hemoglobin, every 1 g/dL decrease	1.099	0.954–1.266	0.190
Serum albumin, every 1 mg/dL increase	0.480	0.280–0.830	0.008
FBS, every 10 mg/dL increase	1.042	0.805–1.349	0.753
TG-HDL ratio >3.29	2.88	1.160 – 7.170	0.023

Notes: Cox proportional hazards regression; the full multivariable model included all covariates with univariable $p < 0.10$.

Supplementary Table S2. Comparison of Hazard Ratios for Cardiovascular Mortality by Different Lipid Ratios (primary TG/HDL-C cut-off = 3.29).

Lipid Parameters	AUC	HR	95% CI	p-value
TG/HDL ratio	0.77	1.25	1.050 – 1.450	0.015
TC/HDL ratio	0.62	1.18	0.990 – 1.320	0.070
LDL/HDL ratio	0.58	1.10	0.950 – 1.280	0.150

TG	0.69	1.02	1.000-1.100	0.019
HDL	0.72	0.82	0.540–1.030	0.052

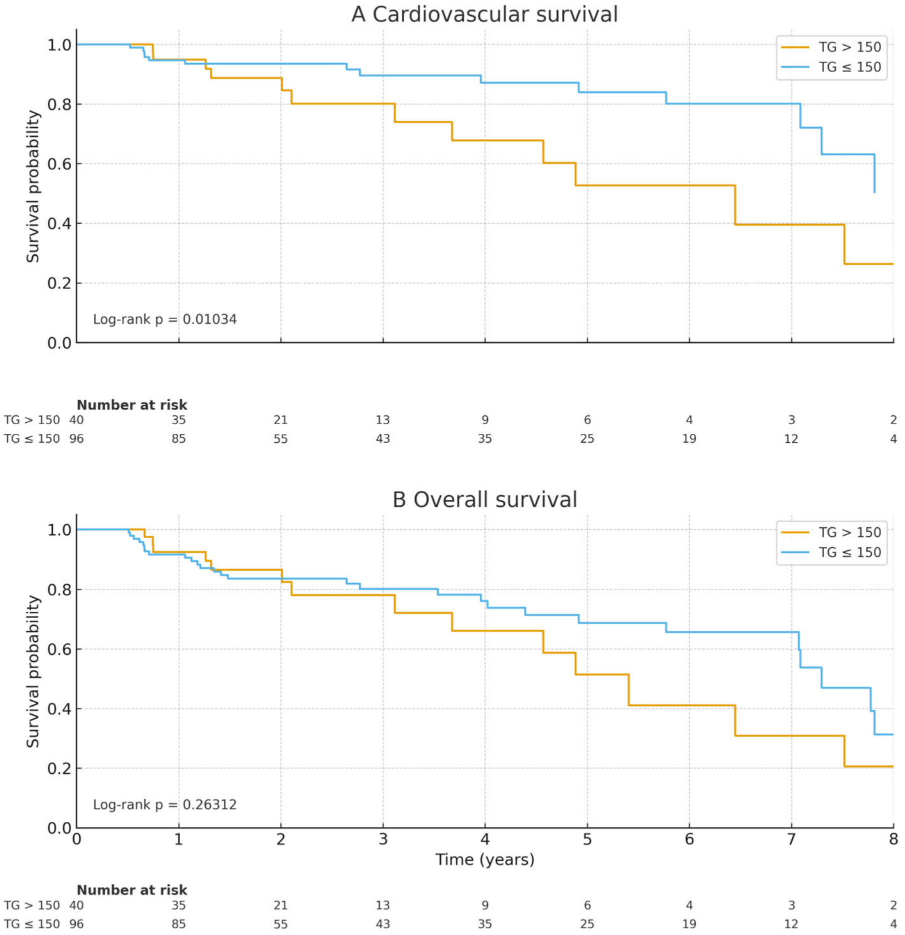

Supplementary Figure S1. Kaplan–Meier curves showing cardiovascular and overall survival according to serum triglyceride levels (>150 mg/dL vs. ≤150 mg/dL).

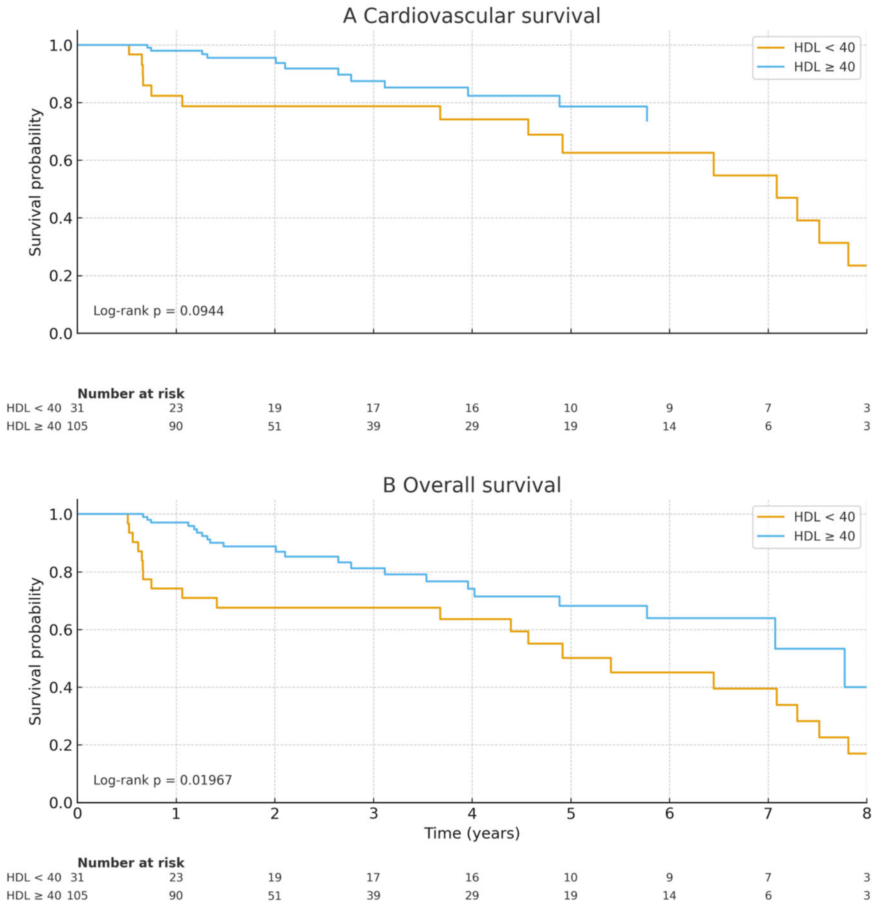

Supplementary Figure S2. Kaplan–Meier curves showing cardiovascular and overall survival according to serum HDL-C levels (<40 mg/dL vs. ≥40 mg/dL).
